# Perceptions and Attitudes Toward Telemedicine by Clinicians and Patients in Japan During the COVID-19 Pandemic

**DOI:** 10.1089/tmr.2021.0012

**Published:** 2021-07-19

**Authors:** Rie Wakimizu, Haruo Kuroki, Katsumi Ohbayashi, Hiroki Ohashi, Kazue Yamaoka, Ai Sonoda, Shinsuke Muto

**Affiliations:** ^1^ Division of Health Innovation and Nursing, Faculty of Medicine, Department of Child Health Care Nursing, University of Tsukuba, Tsukuba City, Japan.; ^2^ Sotobo Children's Clinic, Chiba, Japan.; ^3^ Ohbayashi Clinic, Tochigi, Japan.; ^4^ TAMA Family Clinic, Tokyo, Japan.; ^5^ Teikyo University Graduate School of Public Health, Tokyo, Japan.; ^6^ Tetsuyu Institute Medical Corporation, Tokyo, Japan.; ^7^ Integrity Healthcare Co., Ltd. Tokyo, Japan.

**Keywords:** attitude, COVID-19 pandemic, Japan, perception, telemedicine

## Abstract

***Background:*** Coronavirus disease 2019 (COVID-19) has spread rapidly worldwide. In Japan, the spread of COVID-19 was recognized and a state of emergency declared in April 2020. In response, public health interventions, such as discouraging people from leaving their homes unnecessarily, were enacted across the country. Under these circumstances, telemedicine has received a great deal of social attention, and it has become necessary to identify the perceptions of and attitudes toward telemedicine by clinicians and patients and to clarify the problems and advantages.

***Materials and Methods:*** Ten clinicians and 10 family members (if the patient was pediatric or elderly, a caregiver was included) were invited to participate in individual private interviews in 2020. All interviews were conducted from October to December 2020 using a semistructured interview guide. All transcripts were coded using thematic content analysis.

***Results:*** Four categories from clinicians and five from patients were identified as perceptions of and attitudes toward telemedicine. Both evaluated the usefulness and convenience of telemedicine in the same manner, but there was a large gap in the content under the safety and problem categories.

***Discussion:*** It is necessary to disseminate information about the communication techniques unique to telemedicine to doctors and to improve the “operation and introduction” and “communication environment and device settings” when starting or using telemedicine for all patients.

***Conclusions:*** The perceptions and attitudes identified in this study will be useful for establishing and developing a telemedicine system in Japan.

## Introduction

### Background

Coronavirus disease 2019 (COVID-19) was first detected in Wuhan (Hubei province, China) in December 2019 and since then has rapidly spread globally with the World Health Organization declaring COVID-19 a pandemic. To address the spread of COVID-19, Japan declared a state of emergency in April 2020. Subsequently, public health interventions, such as discouraging people from leaving their homes unnecessarily, were conducted across the country. Under such circumstances, telemedicine has received a great deal of social attention. In utilizing telemedicine, it is important to provide continuous medical care while clarifying the perceptions and attitudes of both health care professionals and patients. This concern has been discussed in the national assembly.

The spread of COVID-19 has significantly changed health care. One of the major changes is the popularization of telemedicine,^1,2^ which has become prevalent in many parts of the world facing the spread of COVID-19 because of its improved access and remote treatment. Telemedicine is expected to become widespread as a new form of patient-oriented medical care for medical professionals and patients/residents.^3–5^

However, it is important to evaluate both doctors' and patients' experiences of and feelings regarding telemedicine in medical treatment and consultation.^6–8^ Qualitative research methods, such as carefully prepared semistructured interviews and analyses of verbatim records, are useful for investigating people's experiences, perceptions, and attitudes.^9,10^

This study aimed to gather and assess the perceptions and attitudes of clinicians and patients toward telemedicine and to discuss the telemedicine system from both users' perspectives, especially focusing on accessibility and usability in Japan. We expect that this study would be very useful given the lack of reports on clinician and patient perceptions of and attitudes toward telemedicine in Japan. This is the first qualitative study of interviews with clinicians and patients in Japan, making it novel research. In this study, “telemedicine” is defined as “synchronous medical consultations using video chat.”

## Materials and Methods

### Participants and recruitment

The study involved 10 clinicians and 10 patients, including a caregiver if the patient was a child or an elderly person who had used telemedicine. “Regarding timely and special handling of medical treatment using information and communication equipment when the new coronavirus infection spreads,”^11^ we recruited the participants through snowball sampling. Clinicians and patients were included as long as they had telemedicine experience. First, we recruited one acquaintance who had experience with telemedicine. Further participants were recruited from different hospitals or clinics in both urban and rural areas and including both public and private institutions. No reward was distributed to the participants.

### Method and content of the interviews

The research manual and consent form were sent to each participant from the research office. When consent was obtained through the forms, the researcher scheduled interviews, which were later conducted over the telephone or Zoom. We used an interview guide to gather respondents' perceptions of and attitudes toward telemedicine. All interviews were conducted between October and December 2020. We recorded all interviews and verbatim records.

### Content analysis

The narrative element of respondents' perceptions of and attitudes toward telemedicine was extracted by the first author to avoid impairing the context. Each narrative element was developed into a short sentence (data) that expressed the meaning and content in a simple manner. At regular meetings once a week, all the authors confirmed that the meaning of each narrative element for a short sentence (data) was preserved. These data were then compared and classified according to the similarity of their meaning and content, and data with a common meaning or content were collected and coded. Furthermore, subcategories were organized by meaning, and the degree of abstraction was increased by comparing them with other categories. The codes and subcategories were expressed using a sentence that clearly showed the meaning. In interpreting the data, we were supervised by five telemedicine practitioners and two public health professionals to ensure validity. In the data analysis stage, to ensure credibility, all authors were involved in discussions at each stage of the data extraction and subcategory- and category-naming processes.

### Ethical consideration

This study was approved by the Institutional Review Board of Tetsuyu Institute Medical Corporation (Approval No. 20-4-2).

## Results

### Attributes of clinicians

The average interview duration was 39 min and 43 sec ±7 min and 10 sec (range 28 min and 17 sec to 48 min and 51 sec). All participants were engaged in telemedicine, and the introduction period was from 2016 to 2020 (Table 1). There was one participant in their 30s, two in their 40s, five in their 50s, and two in their 60s; the working areas were Tohoku, Kanto, Chubu, Kansai, and Kyushu. Six individuals worked in each clinic, and four worked in each hospital. Information on the number of beds owned by each hospital is presented in Table 1.

**Table 1.** Clinicians Interviewed About Telemedicine

ID	Workplace and number of beds	Department	Gender	Age (years)	Clinical experience (years)	Telemedicine practice commencement date	Location
1	Clinic	Internal Medicine	M	50s	29	May 18	Chubu
2	Hospital (271 beds)	Psychiatry	M	60s	36	August 20	Kanto
3	Clinic	Pediatrics	F	50s	25	April 18	Kansai
4	Clinic	OBGYN	F	60s	37	September 16	Kansai
5	Hospital (350 beds)	General Medicine/Neurosurgery	M	50s	30	February 20	Kanto
6	Hospital (111 beds)	Home Healthcare	M	30s	14	April 20	Kyushu
7	Clinic	ENT	M	50s	28	December 17	Kanto
8	Clinic	Home Healthcare	M	50s	26	April 20	Kanto
9	Hospital (60 beds)	Orthopedic Surgery	M	40s	23	December 18	Tohoku
10	Clinic	Internal Medicine/Psychiatry	M	30s	7	April 20	Kanto

### Clinicians' perceptions of and attitudes toward telemedicine during the COVID-19 pandemic

We extracted four main categories as clinicians' attitudes toward or perceptions of the use of telemedicine, which are reported as follows at the subcategory level. First, 13 subcategories and 58 codes were extracted as the effectiveness of telemedicine. The contents of the subcategories are listed in Table 2. Second, 13 subcategories and 96 codes were extracted as the safety of telemedicine. In this study, “safety” is defined as “the degree of safety (the state in which the risk is suppressed to an acceptable level) regarding the information to be handled and the medical care to be provided.” Safety here also refers to a state in which individuals and the general public are limited in the extent to which they can tolerate harm, such as security incidents and medical accidents. The contents of these subcategories are listed in Table 3. Third, the responses to the problems with telemedicine itself as well as profitability and system operations were composed of 3 subcategories and 26 codes and 8 subcategories and 21 codes, respectively. The contents of the subcategories are listed in Table 4. Finally, related to the prospects and requests regarding telemedicine, 6 subcategories and 24 codes were extracted as prospects and requests. The contents of the subcategories are listed in Supplementary Table S1.

**Table 2.** Effectiveness of Telemedicine as Perceived by Clinicians

• Telemedicine is a form of medical care and an infection–prevention measure that meets patients' growing needs (*N* = 10).
• Telemedicine visits are effective as first contact and are suitable for patients who want a chance to visit (*N* = 3).
• Even if clinicians cannot make a definitive diagnosis in the first telemedicine session, they can decide to meet patients face-to-face for treatment (*N* = 8).
• If telemedicine includes video technology, a large amount of information is exchanged, so effective medical consultation becomes possible (*N* = 5).
• Telemedicine lowers the threshold for patient visits and raises medication adherence (*N* = 3).
• Clinicians can take control of their treatment process and feel relieved because busy office workers and other people who used to give up going to the hospital would revisit if they could use telemedicine (*N* = 3).
• The burden of going to the hospital is reduced for both elderly individuals and the family members that accompany them (*N* = 4).
• Patients can seek and reach clinicians who can speak with peace of mind, whether they are family doctors or not, even if they are far away (*N* = 2).
• Clinicians can take a closer look at patients' private settings (*N* = 3).
• Telemedicine has a strong counseling element, so clinicians can communicate intimately with patients (*N* = 6).
• Telemedicine is effective if the patient has a stable chronic disease (*N* = 10).
• Patients with a stable chronic disease can be effectively followed up with telemedicine except when patients are in a psychiatrically unstable condition (*N* = 10).

The number of clinicians/patients who actually contributed to the topic/theme.

**Table 3.** Safety of Telemedicine as Perceived by Clinicians

• Whether it is possible to provide patients with proper information and take appropriate steps is the key to safety (*N* = 10).
• Information must be obtained in advance to make a certain diagnosis for a first-visit patient with telemedicine, but it is also important to be able to extract the information while seeing the patient (*N* = 4).
• Clinicians are aware of problems facing telemedicine in terms of complying with security and privacy standards (*N* = 10).
• Clinicians should educate patients on the safe use of telemedicine (*N* = 6).
• Telemedicine can minimize contact between patients and staff and provide safe medical care without causing nosocomial infections (*N* = 10).
• Since palpation and checkup are not possible and little information is provided, there are symptoms and condition images that are difficult to definitively diagnose, and they feel the limits of telemedicine from the viewpoint of safety (*N* = 6).
• First-time patients are at high risk if they are examined through telemedicine and are not accepted at some hospitals (*N* = 2).
• Telemedicine can be safely performed only for patients with limited chronic illness (*N* = 10).
• Patients with some chronic illnesses in a stable condition can be seen with telemedicine, but those with sudden symptoms, poor control, heading in the wrong direction, or serious illness may be overlooked if not tested, so they encourage patients to engage in immediate face-to-face treatment (*N* = 10).
• The use of telemedicine by elderly individuals alone is risky from the perspective of safety (*N* = 3).
• Immediate face-to-face treatment is needed for patients with symptoms such as hallucinations, delusions, and suicidal ideation (*N* = 1).
• They feel anxiety and hesitation regarding whether the appropriate medication has been prescribed (*N* = 2).
• They encourage face-to-face medical care for patients who use telemedicine as if it were a prescription pharmacy (*N* = 1).

The number of clinicians/patients who actually contributed to the topic/theme.

**Table 4.** Problems with Telemedicine as Perceived by Clinicians

“Profitability”
• It is not profitable in terms of management (*N* = 10).
• Additional costs were incurred due to the need for personnel to explain its use (*N* = 4).
• Disease restrictions and the control of medical scores in telemedicine should be reviewed (*N* = 7).
“System operations”
• They were confused about communication problems, image quality limits, and how to use the device (*N* = 3).
• It takes time and effort to teach the patient how to use the device (*N* = 8).
• They are worried about the manners and literacy of the users (*N* = 5).
• They are confused by the sensitivity complaints from patients regarding operations, not medical treatment through telemedicine (*N* = 4).
• They struggle with effective operation due to regional issues related to medical use and aging users (*N* = 3).
• It is difficult to increase the quota and numbers of telemedicine because they are exhausted from the dual work of telemedicine and face-to-face medical care (*N* = 6).
• It is difficult to collaborate with other medical occupations (*N* = 4).
• The clinicians around them are not trying to adopt telemedicine (*N* = 3).

The number of clinicians/patients who actually contributed to the topic/theme.

### Attributes of patients

The average duration of the interviews with the participants was 27 min and 56 sec ±5 min and 32 sec (range 18 min and 00 sec to 37 min and 50 sec). All participants were using telemedicine, and the introductory period was from 2016 to 2020 (Table 5). The departments they visited were mainly internal medicine, and their main symptoms were migraines, hypertension, chronic rhinitis, and constipation. Their ages ranged from 6 to >70 years, but their area of residence was limited to the Kanto region. Four caregivers responded on behalf of the patients.

**Table 5.** Patients and Their Families Interviewed About Telemedicine

ID	Department	Gender	Age (years)	Main symptoms, ADL, etc.	Telemedicine use start date	Number of telemedicine visits	Area of residence
1	Internal Medicine	F	40s	Migraine	July 20	2	Kanto
2	Internal Medicine	F	30s	Migraine	May 20	5	Kanto
3	Pediatrics	F	20s	Allergic rhinitis, high fever in April	April 20	6	Kanto
4	Pediatrics	Pediatric patient: M; Parent: F	Pediatric patient: 7; Parent: 40s	Mitochondrial disease, vomiting and hypoglycemia symptoms, total blindness	March 20	11	Kanto
5	Neurosurgery/Internal Medicine/Rehabilitation Medicine	Patient: M; Biological daughter: F	Patient: 70s; Biological daughter: 50s	High blood pressure, sequelae of stroke, wheelchair (cane) dependent	March 19	12	Kanto
6	Internal Medicine	M	40s	Asthma, eosinophilic otitis media	July 5	21	Kanto
7	Internal Medicine	M	40s	Migraine, high blood pressure, heart attack, sleep apnea syndrome (nighttime CPAP use)	May 20	12	Kanto
8	Internal Medicine	F	70s	High blood pressure	May 20	4	Kanto
9	Internal Medicine/Neurosurgery	M	70s	High blood pressure, sequelae of stroke, wheelchair (cane) dependent	May 20	4	Kanto
10	Pediatrics	Pediatric patients: M; Parent: F	Pediatric patients: 6 and 11; Parent: 30s	Allergic rhinitis, ADHD, constipation	July 5	7	Kanto
11	Pediatrics	Pediatric patient: F; Parent: F	Pediatric patient: 3; Parent: 30s	Chronic constipation	April 20	7	Kanto

ID8 and ID9 are husband and wife (living together).

ADHD, attention-deficit/hyperactivity disorder; ADL, activities of daily living; CPAP, continuous positive airway pressure.

### Patient's and caregivers' perceptions of and attitudes toward telemedicine during the COVID-19 pandemic

We extracted five main categories of patients' and caregivers' attitudes toward or perceptions of the use of telemedicine, which are reported as follows at the subcategory level. First, 8 subcategories and 17 codes were extracted as the triggers and reasons for deciding to use telemedicine. The contents of the subcategories are listed in Supplementary Table S2. Second, the responses to the effectiveness of telemedicine itself as well as the proper use of telemedicine and face-to-face medical care comprised 6 subcategories and 15 codes and 2 subcategories and 16 codes, respectively. The contents of these subcategories are listed in Table 6. Third, two subcategories and five codes were extracted as safety in the use of telemedicine as recognized by patients and their families. The contents of the subcategories are listed in Supplementary Table S3. Fourth, regarding problems related to using telemedicine, 5 subcategories and 22 codes related to “examination and prescription,” “current communication environment and device settings,” and “operation and introduction” were extracted. The contents of the subcategories are listed in Table 7. Finally, related to the prospects and requests regarding telemedicine, 5 subcategories and 24 codes were extracted as prospects and requests. The contents of the subcategories are listed in Supplementary Table S4.

**Table 6.** Benefits of Using Telemedicine as Perceived by Patients and Their Families

“Effectiveness of telemedicine”
• They avoid getting infected with COVID-19 or influenza (*N* = 11).
• With telemedicine, there is no waiting time, there is no waste of time, and it is possible to receive a medical examination in one's spare time (*N* = 11).
• If one's symptoms are chronic and stable, they can be conveniently treated and managed without any problems (*N* = 11).
• For unexpected symptoms, they can rest assured that preventive drugs will be prescribed through telemedicine as needed (*N* = 11).
• The burden of going to the hospital is reduced for both the patient and their family (*N* = 11).
• It was helpful to be able to use telemedicine when they were having severe symptoms and did not go directly to a medical institution (*N* = 2).
“Proper use of telemedicine and face-to-face medical care”
• If the patient's symptoms remain stable and they merely want to be prescribed drugs, they can choose telemedicine (*N* = 11).
• If their symptoms are unusual, they are sick, they need a test, or they cannot judge or identify the issue themselves, they can cancel telemedicine and choose face-to-face care (*N* = 10).

The number of clinicians/patients who actually contributed to the topic/theme.

COVID-19, coronavirus disease 2019.

**Table 7.** Problems in Using Telemedicine as Perceived by Patients and Their Families

“Examination and prescription”
• Unlike in face-to-face medical care, they felt anxious about medical examination, diagnosis, and drug prescription through telemedicine (*N* = 3).
• They find it inconvenient to obtain prescriptions and receive medicine through telemedicine (*N* = 5).
“Current communication environment and device settings”
• It was difficult to understand how to use and operate the device (*N* = 6).
• Patients had difficulty showing their throat and nose through the screen, doctors might have had difficulty seeing them, and hearing was poor as the audio was interrupted (*N* = 4).
“Operation and introduction”
• It is becoming more difficult to make a reservation (*N* = 3).
• Only some medical institutions have introduced telemedicine (*N* = 8).

The number of clinicians/patients who actually contributed to the topic/theme.

## Discussion

### Clinicians' perceptions of and attitudes toward telemedicine

Before the spread of COVID-19, Japanese clinicians considered it a matter of course that patients would visit medical facilities for examinations. However, in this study, clinicians pointed out the usefulness and safety of telemedicine during the pandemic; specifically, they found that they could “avoid the risk of infection and offer patients non-face-to-face examinations.” Clinicians stated that, although face-to-face examinations are essential when symptoms worsen, it is possible to examine and treat patients during the initial visits through visual examinations and interviews using telemedicine, demonstrating a change in attitude. Furthermore, clinicians believed that a patient's ability to receive treatment from a specialist of their choice, regardless of their family doctor or area of residence, makes telemedicine effective. There are certain diseases and patient conditions for which telemedicine is appropriate.^12^ Clinicians in this study recognized that “telemedicine is effective if the patient has a stable chronic disease” and “telemedicine has a strong counseling element, so clinicians can communicate intimately with patients,” and they discussed experiences with telemedicine in which they were able to hear deeper concerns from patients about their diseases and treatments that had not been discussed in the outpatient examination room in the past.

Clinicians perceived the safety of telemedicine during the COVID-19 pandemic first as “minimizing the risk of human contact and infection through a non-face-to-face examination system” and second as “ensuring the safety of patient information handled before and after examinations, maintaining patient compliance during treatment, and educating patients for health literacy.” The majority of clinicians treated patients with the following clear-cut policy: “Patients with some chronic illnesses in a stable condition can be seen with telemedicine, but those with sudden symptoms, poor control, heading in the wrong direction, or serious illness may be overlooked if not tested, so they encourage patients to have immediate face-to-face treatment.” It is essential to promote education about the safe use of telemedicine for both patients and caregivers.

Clinicians felt that it was dangerous to prescribe medication using telemedicine. They thought that, without certain restrictions, this could be the same as online shopping for medication and that detailed guidelines for prescribing medication are essential. This also leads to the issue of the centralization and evaluation of personal health records and other medical information,^13,14^ and, thus, a system and set of rules for telemedicine in Japan that would ensure safe prescriptions, examinations, and handling of information should receive immediate attention.

Concerns were largely divided into those regarding profit and those regarding operations. In Japan, compensation for telemedicine is lower than that for face-to-face examinations. Concerning profit, clinicians recognized that “it is not profitable in terms of management” and that “additional costs were incurred due to the requirement for personnel who provided explanations” and discussed this as one factor hindering the popularization of telemedicine. Regarding operations, the concern “about the manners and literacy of the users” was identified, and clinicians saw patients canceling without notice as a major operational issue under the current system, in which a scheduling fee cannot be charged for insurance medical services. This demonstrates the need to educate patients on how to use telemedicine. Simultaneously, there were many reports of patient complaints that their telemedicine appointments' start time was delayed because appointments fell behind schedule. Clinicians were also found to be burdened by simultaneously engaging in face-to-face examinations and telemedicine. It was suggested that this burden could be reduced by establishing a system in which trained office staff are responsible for time management and paperwork, and clinicians are only responsible for examinations. This study also revealed that clinicians are at a loss because instruction and communication with patients, which are easy in face-to-face settings, do not work for telemedicine. This is thought to be directly related to the effectiveness and safety of the examinations. Thus, it is necessary to develop and disseminate educational videos explaining examination techniques specific to telemedicine for Japanese clinicians as soon as possible.

### Patients' and families' perceptions of and attitudes toward telemedicine

All participants stated that they felt anxious about visiting a medical facility for a face-to-face examination during the COVID-19 pandemic. Telemedicine use primarily began between April and July of 2020, a period during and immediately after Japan's state of emergency declaration, indicating a decision to switch from face-to-face to online care to avoid potential COVID-19 infections. A previous survey also found significantly higher rates of anxiety about visiting a hospital during the COVID-19 pandemic among telemedicine users compared with nonusers.^15^ In general, telemedicine was supported by Japanese patients and their families as a tool for avoiding the risk of being infected with COVID-19.

Patients mentioned the lack of time spent commuting to the hospital or waiting as one of the “benefits of using telemedicine,” whereas family members discussed being relieved of the hassle of traveling to the hospital and accompanying the patient. Most participants in this study were currently taking some sort of medication and recognized the effectiveness of telemedicine with the primary goal of prescribing drugs in cases in which there were no changes in symptoms. They were also aware that they could receive a face-to-face examination by a clinician in the event of worsening symptoms. A previous survey^15^ found that mothers were hesitant to use telemedicine because they felt that their children would receive a more accurate examination at a face-to-face appointment. It is necessary for clinicians to explain to all users that they can switch to face-to-face care immediately if their symptoms change or if their condition deteriorates.

The disadvantages of telemedicine include concerns that clinicians cannot perform palpation and have a higher chance of overlooking symptoms or misdiagnosis compared with face-to-face examinations because insufficient information makes it impossible for the clinician to accurately grasp the patient's condition. Although we can expect that communication technologies will continue to advance, past research^16^ has argued that telemedicine has significant disadvantages in the examination and treatment of patients for whom the doctor–patient relationship has been damaged. Building trusting relationships between clinicians and patients helps patients recognize that they can feel the same sense of security with telemedicine as they do with face-to-face care and leads to an increased desire to use telemedicine services. Simultaneously, the results of the interviews in this study suggested that, when necessary, patients and their families used telemedicine even without a long-term trusting relationship with the clinician. Thus, a trusting relationship with the clinician is not a prerequisite for all patients. As past research shows,^17^ to expand telemedicine in Japan, decisions regarding whether to use telemedicine or face-to-face services must be made appropriately according to both the patient's disease and their condition at the time.

Johansson et al.^18^ reported two primary reasons why patients living in rural areas of Sweden did not choose telemedicine: (1) they wanted to be examined by a clinician directly, and (2) they did not know how to use communication devices. These findings are consistent with those of this study. To aid the dissemination of telemedicine in the future, it is necessary to establish a system in which even patients who are not accustomed to using communication devices are able to utilize telemedicine. Specifically, as patients stated that they were confused about which buttons they should press when they started to use such services on an unfamiliar device, it is suggested that medical staff demonstrate for patients (particularly older adults) and their families how to actually use the services. Despite recognizing several issues, patients and their families perceived more “benefits of using telemedicine,” which is likely to lead to its continued use. In addition, all participants in this study wanted to continue using telemedicine, and their “prospects and requests regarding telemedicine” included that automatic payment after telemedicine appointments, electronic prescriptions, medication delivery, and online medication instructions be provided together as a package. Thus, it is necessary to strive to improve patient satisfaction with these services.

This study is limited in that only clinicians and patients with experience using telemedicine were interviewed. In addition, as study limitations, we added the small sample sizes in both groups in this study and the lack of data on actual outcomes in this article. Although past studies reported the status of telemedicine use by the families of pediatric patients,^15,19–21^ this study offers significant new findings as we were able to identify perceptions of effectiveness and safety, problems, and prospects of telemedicine among doctors and patients of all ages. Going forward, multimethod longitudinal studies using larger and more diverse samples will be necessary to periodically explore the opinions of telemedicine users.

## Conclusion

The spread of COVID-19 has driven increased telemedicine utilization across Japan, necessitating the analysis of the perceptions of and attitudes toward telemedicine by clinicians and patients conducted in this study. Some perceptions and attitudes were the same, whereas others differed. Communication techniques unique to telemedicine should be related to doctors, and the operation and introduction of systems and the communication environment and device settings should be improved for patients. The findings of this study will be useful for establishing and developing a telemedicine system in Japan.

## Supplementary Material

Supplemental data

Supplemental data

Supplemental data

Supplemental data

## Abbreviation Used

COVID-19: coronavirus disease 2019
