# Supplementary material for: Perceptions and Attitudes Toward Telemedicine by Clinicians and Patients in Japan During the COVID-19 Pandemic
Source: Telemed Rep. 2021 Jul 19;2(1):197–204. doi: 10.1089/tmr.2021.0012 (PMC8812287; doi:10.1089/tmr.2021.0012)
Supplement: Supplemental data [file Supp_TableS1.docx]

**Supplemental Information**

**Table S1. Prospects and requests regarding telemedicine as perceived by clinicians**

| - It would be good if we could establish a cooperative system with secondary hospitals, core hospitals, and neighboring clinics in the same area. (N = 8) |
| --- |
| - It is important to build a follow-up system when the first medical examination of a distant patient is performed via telemedicine. (N = 9) |
| - In the near future, telemedicine may be deployed to all those who are in a facility, such as elderly housing with care and welfare facilities. (N = 4) |
| - Communication and correspondence in telemedicine are not the same as face-to-face care, and it is necessary to disseminate the techniques unique to telemedicine. (N = 5) |
| - It would be good if the current telemedicine system continued and was not just a temporary measure. (N = 10) |
| - It would be good if the telemedicine system could be applied to the consolidation of medical care across regions, especially nighttime medical care. (N = 1) |

(the number of clinicians/patients who actually contributed to the topic/theme)
