# Supplementary material for: Perceptions and Attitudes Toward Telemedicine by Clinicians and Patients in Japan During the COVID-19 Pandemic
Source: Telemed Rep. 2021 Jul 19;2(1):197–204. doi: 10.1089/tmr.2021.0012 (PMC8812287; doi:10.1089/tmr.2021.0012)
Supplement: Supplemental data [file Supp_TableS2.docx]

**Table S2. Reasons for using telemedicine as perceived by patients and their families**

| “Triggers” |
| --- |
| - Recommended by the family doctor. (N = 9) |
| - Due to the COVID-19 pandemic, some people started working from home and could not attend the clinic they used to go to. (N = 1) |
| - Some people had a fever but were not allowed to go to a medical facility due to the prevalence of COVID-19. (N = 1) |
| “Reasons” |
| - There was a relationship of trust with the family doctor. (N = 9) |
| - Some people had to be prescribed medicine due to aggregated symptoms. (N = 10) |
| - With telemedicine, they can have a medical examination in their spare time, thus saving time. (N = 10) |
| - They can avoid getting infected with COVID-19 or the flu. (N = 11) |

(the number of clinicians/patients who actually contributed to the topic/theme)
