# Supplementary material for: Perceptions and Attitudes Toward Telemedicine by Clinicians and Patients in Japan During the COVID-19 Pandemic
Source: Telemed Rep. 2021 Jul 19;2(1):197–204. doi: 10.1089/tmr.2021.0012 (PMC8812287; doi:10.1089/tmr.2021.0012)
Supplement: Supplemental data [file Supp_TableS3.docx]

**Table S3. Safety of using telemedicine as perceived by patients and their families**

| - Time was not taken to explain privacy and security for medical professionals, and they do not really care about information leaks. (N = 9) |
| --- |
| - Regarding the handling of personal information online, they had the doctors explain to them each time they were concerned, so they have not had to worry so far. (N = 11) |

(the number of clinicians/patients who actually contributed to the topic/theme)
