# Supplementary material for: Perceptions and Attitudes Toward Telemedicine by Clinicians and Patients in Japan During the COVID-19 Pandemic
Source: Telemed Rep. 2021 Jul 19;2(1):197–204. doi: 10.1089/tmr.2021.0012 (PMC8812287; doi:10.1089/tmr.2021.0012)
Supplement: Supplemental data [file Supp_TableS4.docx]

**Table S4. Prospects and requests regarding telemedicine as perceived by patients and their families**

| - It would be good if costs such as communication charges and system charges were reduced. (N = 7) |
| --- |
| - It would be good if the method of receiving the medicine was examined and new services such as an immediate delivery service for medicine were started. (N = 9) |
| - It would be nice if there were no national institutional restrictions on the amount of medication prescribed at one time. (N = 9) |
| - To promote the dissemination of telemedicine across a wide range of participants and generations, it is desirable to have careful assistance with tools and people regarding how to use and operate the device.　(N = 4) |
| - It would be nice if telemedicine could be used permanently rather than for a limited time. (N = 11) |

(the number of clinicians/patients who actually contributed to the topic/theme)
